# Review of intravenous immunoglobulin replacement therapy trials for primary humoral immunodeficiency patients

**DOI:** 10.1007/s15010-012-0323-9

**Published:** 2012-09-12

**Authors:** H. W. Schroeder, C. J. Dougherty

**Affiliations:** 1 Department of Medicine, Division of Clinical Immunology and Rheumatology, The University of Alabama at Birmingham, Birmingham, AL USA; 2 Clinical and Regulatory Affairs Department, Biotest Pharmaceuticals Corporation, Boca Raton, FL USA

**Keywords:** Intravenous immunoglobulin, IVIG, Primary immunodeficiency, PID, Clinical trial

## Abstract

An available supply of intravenous immunoglobulin (IVIG) is essential for individuals with primary humoral immunodeficiency. A shortage in 1997 prompted the Food and Drug Administration (FDA) to revise guidelines for the licensure, production, and distribution of new IVIG products, including the standardization of United States clinical trials regarding endpoints for safety, efficacy, and pharmacokinetics. The following review is intended to present current information and results of clinical trials in patients with primary immunodeficiency treated with IVIG products currently licensed or awaiting licensure in the United States. The data presented are compiled from published clinical trials and prescribing information generated by manufacturers.

## Introduction

In the Fall of 1997, increased demand, reduced supply, and product recalls created a shortage of intravenous immunoglobulin (IVIG) in the United States. Key factors in the shortfall in production included issues of regulatory compliance and good manufacturing practices. In response, the Food and Drug Administration (FDA) facilitated production and distribution, shortened the review of lot release protocols, expedited the review of license applications, and streamlined clinical trial design.

At the FDA Blood Products Advisory Committee (BPAC) meeting held in March, 1999 [1], the FDA recommended a change in design for a pivotal clinical trial to evaluate the safety and efficacy of a new IVIG product. The new design involved a prospective, randomized, double-blind, parallel-group, positive-controlled, non-inferiority study in 80 subjects with a documented history of primary immune deficiency, in which the safety and efficacy of the test product was to be compared head-to-head to a US-licensed IVIG product. Investigational new drug (IND) sponsors would evaluate efficacy by comparing the serious infection rate in each randomization group over an observation period of 12 months.

Following the March 1999 BPAC meeting, the FDA increasingly recognized several problems with this trial design, e.g., limited numbers of patients that could be recruited for trials, multiple new IVIG products to be tested, and the potential for IVIG shortages. At the March 2000 BPAC meeting [2], the FDA presented an alternate clinical trial design for the evaluation of IVIG safety and efficacy in primary immune deficiency (PID). The new proposal was a single-arm, 12-month, open study of approximately 50 PID patients with safety and efficacy targets based on previous trials. Pharmacokinetic studies would be performed on at least 20 PID patients and observed values should not be inferior to those previously determined for approved products.

Since 1999, the FDA has issued two guidance documents for clinical trials of IVIG to support the marketing of IVIG as replacement therapy for primary humoral deficiency [3, 4]. The 2005 FDA Guidance [3] defined the primary efficacy endpoint as the rate of acute serious bacterial infections (SBIs) during the year of treatment with the study IVIG. Based on the FDA’s examination of historical data, the SBI rate was to be <1.0 per subject per year at the 0.01 level of significance. SBIs were defined as bacteremia/sepsis, bacterial meningitis, osteomyelitis/septic arthritis, bacterial pneumonia, and visceral abscess. Essential diagnostic features for each infection were described.

Safety endpoints were the overall incidence of adverse events (AEs) (regardless of causality) that occurred during or within 1, 24, and 48 h after infusion of the test product. Temporally associated AEs are those that occurred during infusion and up to 48 h after infusion. A primary endpoint was to record the proportion of infusions with one or more temporally associated AEs (including AEs that were determined to be unrelated to the product). The target for this endpoint was an upper one-sided 95 % confidence limit of <0.40 [3]. The number of subjects to achieve the suggested endpoints was estimated at 40–50 subjects.

The 2008 FDA Guidance [4] extended the time period for temporally associated AEs to 72 h and added instructions for conducting pharmacokinetic studies in pediatric patients.

The effect of the 2005 and 2008 Guidances has been to standardize endpoints for safety, efficacy, and pharmacokinetics in clinical trials conducted in the United States over the last decade. We report here the results of clinical trials in patients with primary immunodeficiency treated with IVIG products currently licensed in the United States.

## Early studies

In 1890, Emil Behring and Shibasaburo Kitasato discovered that the injection of guinea pigs with sterilized cultures of diphtheria or tetanus bacilli produced substances in the sera that could neutralize diphtheria or tetanus toxins. They further showed that sera produced in one animal could be used to cure infected animals and to protect healthy animals from diphtheria or tetanus infection mortality [5, 6].

In the 1930s, Enders showed that normal human serum could prevent measles [7]. Contemporary attempts to concentrate this activity in the gamma globulin fraction often resulted in bacterial contamination and adverse reactions in recipients [7]. The use of cold ethanol fractionation as a means to isolate gamma globulin from human plasma finally provided a consistently safe material for injection [7–9]. This material, Cohn Fraction II, was shown by Enders to contain the majority of anti-measles activity [7].

In 1952, Bruton treated a child that had undetectable gamma globulin levels and recurring pneumococcal infections with subcutaneous infusions of human immune serum globulin (ISG, i.e., Cohn Fraction II formulated at 165 mg/mL). The injection of 3.2 g/month ISG increased circulating gamma globulin levels and completely eliminated pneumococcal infections [10]. These observations were rapidly confirmed [7] and treatment with human IgG soon became the standard of care for patients with primary antibody deficiencies [7].

Early clinical studies of ISG were conducted primarily in children who suffered from recurrent bacterial infections, exhibited evidence of deficient antibody production, and had extremely low levels of circulating gamma globulin [11]. ISG was restricted to intramuscular injection because intravenous injection caused severe pyrogenic and cardiovascular reactions in many recipients [12]. In response, a series of manufacturing changes designed to reduce the incidence of side effects led to the development of immune globulins intended for intravenous injection (IVIG).

Spontaneous complement activation (anti-complement activity) by IgG aggregates was considered to be the principal cause of adverse reactions [13]. To suppress anti-complement activity, some manufacturers treated Cohn Fraction II with enzymes or chemical modification. Unfortunately, these treatments also reduced important antibody biological activities required for clinical efficacy and shortened IgG circulating half-lives [14–16].

The recognition that Cohn Fraction II contained trace amounts of highly active contaminants such as prekallikrein activator, prekallikrein, and activated coagulation factors led to the development of Cohn Fraction II purification procedures using anion (DEAE) exchange chromatography. The first DEAE-purified IVIG contained none of the trace contaminants associated with AEs and some antibody biological activities, such as bacterial opsonization and virus neutralization, were higher than in “treated” products [17]. Most commercial IVIGs are now produced with an anion exchange chromatography step and contain relatively low levels of trace contaminants.

In the 1980s, the licensure of IVIG in the United States was generally based on data from relatively small numbers of PID patients. The first IVIG licensed in the United States was chemically modified. Twenty patients with X-linked agammaglobulinemia (XLA, *n* = 9) or common variable immunodeficiency (CVID, *n* = 11) were randomized to receive either 150 mg/kg/4 weeks of IVIG or 100 mg/kg/4 weeks of ISG. Fourteen patients were treated with IVIG for 242 treatment months. This group developed 0.103 acute infections/month of treatment. In contrast, 13 patients treated with ISG for 193 treatment months developed 0.295 acute infections/month of treatment [18].

Cunningham-Rundles et al. [19] studied 21 patients who were treated with 300 mg/kg IVIG every 3 weeks for 1 year following a year of treatment with ISG. In the 18 patients who completed a year of IVIG treatment, the average IgG trough level was 505 mg/dL, compared to 262 mg/dL during ISG treatment. Efficacy endpoints such as days of illness from infection (e.g., upper respiratory tract infections, bronchitis, sinusitis, etc.) were significantly lower (*p* < 0.001) during the IVIG treatment year compared to the ISG treatment year. Adverse reactions to IVIG were observed in 10 of 407 infusions (2.5 %) and were eliminated by slowing or temporarily stopping the infusion [19].

In a trial of another IVIG, Ochs et al. treated 15 PID patients with 400 mg/kg every 4 weeks for 1 year. Twenty-one adverse reactions were associated with 15 of 341 infusions (4.4 %) during the first 48 h after infusion. The incidence of acute upper respiratory infections was as low or lower than infections reported for other preparations [20].

In a third trial, Roifman and Gelfand compared high- versus low-dose therapy in patients with hypogammaglobulinemia and sinopulmonary disease. High-dose therapy (600 mg/kg every 4 weeks) proved efficacious in reducing symptoms, decreasing the frequency of major and minor infections, and significantly improving pulmonary function. The improvement appeared to correlate with a marked reduction in the isolation of *Mycoplasma*, particularly *Ureaplasma urealyticum*, an important cause of infection in patients with hypogammaglobulinemia [21].

## Recent studies in the United States

The following studies reflect the evolution of FDA Guidance documents for testing the safety and efficacy of intravenous immune globulins to be marketed in the United States.

### Gamunex^®^ 10 %

One IVIG was studied using the 1999 BPAC trial design [22]. Twenty-five centers in the US and Canada enrolled 172 patients with PID who were already receiving IVIG replacement therapy. Patients, aged 1–75 years, were randomly assigned to treatment for 9 months with either investigational IVIG (Gamunex^®^ 10 %, *n* = 87) or the US licensed control IVIG (Gamimune^®^ N 10 %, *n* = 85). Seventy-three per-protocol patients in each treatment group were evaluated for efficacy. The primary endpoint was the proportion of patients with at least one validated acute sinopulmonary infection during the 9-month treatment period. Diagnostic criteria for validated infections were defined prospectively. Secondary endpoints were the incidence of all infections, time to first infection, lung function parameters, infusion-related safety, and viral safety [22].

Patient demographics and disease characteristics were essentially the same in each group (Table 1). Validated infections occurred in 9 (12.3 %) patients treated with Gamunex 10 % and 17 (23.3 %) patients treated with Gamimune N 10 %. AE rates within 48 h were very similar in each group. No serious adverse events (SAEs) were reported. Direct Coombs tests (also known as direct antiglobulin tests or DAT) were transiently positive in 29/87 patients (33 %) in the Gamunex 10 % group and 16/85 patients (19 %) in the Gamimune N 10 % group. No clinical or laboratory evidence of hemolysis was detected in the DAT-positive patients [22]. The IgG half-life was 35 days in patients treated with Gamunex 10 % and 34 days in Gamimune N 10 % recipients [23].

**Table 1:** A summary of efficacy results from recent intravenous immunoglobulin (IVIG) clinical trials performed in the United States

Parameter	Gamunex 10 %	Gamimune N 10 %	Octagam 5 %	Gammagard liquid 10 %	Flebogamma 5 % DIF	Privigen 10 %	Flebogamma 10 % DIF	Gammaplex 5 %	Biotest 10 % IVIG
Subjects (*N*)	PP = 73	PP = 73	46	61	50	80	46	50	63
Age (years), mean (range)	35.1	29.5	31 (6–74)	34 (6–72)	38.9 (15–75)	28 (3–69)	36.8 (6–65)	44.0 (9–78)	41.2 (6–75)
Treatment groups	21 days = 9	21 days = 14	21 days = 19	NR	21 days = 13	21 days = 16	21 days = 16	21 days = 22	21 days = 17
	28 days = 64	28 days = 59	28 days = 27		28 days = 33	28 days = 64	28 days = 30	28 days = 28	28 days = 46
Diagnosis	ITT = 87	ITT = 85							
CVID	46 (53 %)	44 (52 %)	28 (61) %	NR	35 (76.1 %)	59 (73.8 %)	37 (80.4 %)	46 (92 %)	51 (81 %)
XLA			13 (28 %)		10 (21.7 %	21 (26.2 %)	8 (17.4 %)	4 (8 %)	6 (9.5 %)
Hypogam	39 (45 %)	35 (41 %)							
Combined ID	1 (1 %)	5 (6 %)							
Other	1 (1 %)	1 (1 %)	5 (11 %)		1 (2.2 %)		1 (2.1 %)		2 (905 %)
Serious infections, no. of patients (%)	9 (12.3%)^a^	17 (23.3%)^a^	3	0	1	6	1	0	2
Serious infections, no. of patients (%)	0.18	0.43	0.12 (upper 98 % CI = 0.279)	0 (upper 95 % CI = 0.060)	0.012 (upper 98 % CI = 0.112)	0.08 (upper 97.5 % CI = 0.182)	0.025 (upper 98 % CI = 0.133)	0 (upper 99 % CI = 0.101)	0.037 (upper 99 % CI = 0.101)
Other infection rate (no./patient/year)				3.4	1.96	3.55	2.2	3.28	2.6
Missed work/school, days/patient/year (range)			5 (0–22)		13 (0–257)	7.94	3	8.7 ± 34.4	2.28
Hospitalizations, days/patient/year (range)			0.3 (0–6)		0.77	2.31	0.6	0.75	0.21
Unscheduled physician/ER visits, mean/patient/year (range)			2 (0–11)		4.3 (0–34)		2.1	5.55 (0–42.1)	
Therapeutic antibiotics (days/patient/year)					55.6		57.7	47.2	
Prophylactic antibiotics (days/patient/year)					81.1		46.1	7 subjects continuous; 33 courses in 16 patients	
Total antibiotics (days/patient/year)					136.7	87.4	103.8		82
IgG half-life (days)	35	34	41 ± 17	35 (95 % CI = 31–42)	21 days = 30 ± 9 28 days = 32 ± 5		21 days = 34 ± 10 28 days = 37 ± 13	41.1	21 days = 20 ± 4 28 days = 34 ± 11
Trough IgG (mg/dL)	NR	NR	21 days = 986 28 days = 883	960–1,120	21 days = 951 ± 132 28 days = 900 ± 92	884–1,027	NR	21 days = 936–1,240^b^ 28 days = 833–1,140^b^	21 days = 1,076 ± 254 28 days = 943 ± 215

*NR* not reported

^a^Serious infections were defined as “validated” infections and included acute sinusitis, acute exacerbation of chronic sinusitis, or pneumonia according to diagnostic criteria defined prospectively

^b^Median values

### Octagam^®^ 5 %

Octagam^®^ 5 % was the first IVIG studied using the 2000 BPAC trial design. Forty-six PID patients at nine clinical sites were treated for 12 months with 300–450 mg/kg IVIG every 21 days or 400–600 mg/kg every 28 days [24]. The primary efficacy endpoint was the rate of serious infections per patient per year and a requirement that the annual rate be <1 serious infection/patient/year. Serious infections were defined prospectively as pneumonia, bacteremia or sepsis, osteomyelitis or septic arthritis, visceral abscess, and bacterial or viral meningitis.

All 46 patients received at least one infusion of the study IVIG and were included in the study analysis. They ranged in age from 6 to 74 years (mean = 31). The underlying PID diagnoses were CVID in 28 patients (61 %) and XLA in 13 patients (28 %). Nineteen patients received IVIG infusions every 21 days and 27 were infused every 28 days [24].

Three patients experienced five serious infections. One patient had one episode of pneumonia and two infections were recorded as bacteremia or sepsis infection. The remaining two patients experienced one bacteremia or sepsis infection each. The overall serious infection rate was 0.1 serious infection/subject/year. Thirty subjects (65 %) missed one or more days of work or school; four subjects were hospitalized at least once because of pneumonia, severe cellulitis, severe upper abdominal pain, or gastroenteritis, and twenty-seven subjects visited a physician or emergency room (ER) at least once (also see Table 1). The IgG half-life was 41 ± 17 days. As shown in Table 2, 12 SAEs were recorded in six patients. None were related to the study IVIG [24].

**Table 2:** A summary of adverse event (AE) results from recent IVIG clinical trials performed in the United States

Parameter	Gamunex 10 %	Gamimune N 10 %	Octagam 5 %	Gammagard liquid 10 %	Flebogamma 5 % DIF	Privigen 10 %	Flebogamma 10 % DIF	Gammaplex 5 %	Biotest 10 % IVIG
Subjects (*N*)	PP = 73	PP = 73	46	61	50	80	46	50	63
Infusions (*n*)	825	865	654	826	709	1,038	601	703	746
AEs (*n*)	NR	NR	793	896	595	1,330	719	237	937
All AEs (% of infusions)	17.1 %	18.8 %						33.7 %	
Related AEs (% of infusions)	5.7 %	5.5 %	5 %		14.1 %	9 %	27.6 %	16.4 %	
Related AEs (% of subjects)			21 days = 26 % 28 days = 44 %				83 %	48 %	63.5 %
SAEs (related to study IVIG)	NR	NR	0	2 aseptic meningitis episodes in 1 patient	0	5 in 1 patient	0	2	2 in 1 patient
SAEs (unrelated to study IVIG)	NR	NR	12 SAEs in 6 patients (none related to study IVIG)	13 in 7 patients	0	36 in 15 patients	4 in 3 patients	5	9 in 6 patients
Temporally associated AEs (0–72 h) by infusion, *n* (%)	(0–48 h) 17.1 %	(0–48 h) 18.8 %	(0–30 min) 23 %	345 total NR	216 total 30.5 %	397 total (38.2 %)	291 (48.4 %)		431 total
Infusions with ≥1 temporal (72 h) AEs (%)					20.1 % (upper 95 % CI = 24.4 %)	20.8 % (upper 97.5 % CI = 23.8 %)	47.0 %	21.2 % (upper 97.5 % CI = 24.2 %)^a^	27.7 % (upper 95 % CI = 30.6 %)
Most frequent AEs	Headache	Headache	Headache > nausea > chills	Headache > fever > fatigue	Headache > fever	Headache > pain > fatigue	Headache > fever > rigors	Headache > pyrexia > nausea	Headache > fatigue
Coombs-positive subjects (%)	33 %	19 %	NR	NR	NR	46.8 %	23.3 %	8.5 %	NR

*NR* not reported

^a^Includes infections

### Gammagard^®^ Liquid 10 %

Using the same basic study design, Gammagard^®^ Liquid 10 % was evaluated in 61 adults and children with PID in an open-label, historically controlled, single-arm clinical trial. The primary efficacy endpoint was the incidence of acute SBIs per subject per year, with a target of ≤1.0 SBIs per subject per year. SBIs were defined prospectively as bacterial pneumonia, bacteremia/sepsis, osteomyelitis/septic arthritis, visceral abscess, and bacterial/meningitis, all of which were required to meet specific diagnostic criteria. Secondary efficacy endpoints were the rate of predefined non-SBIs that commonly occur in PID patients and the number of hospitalizations resulting from infections [25].

Subjects were to be treated every 21 or 28 days with 300–600 mg/kg of the study IVIG for 12 months. The majority of subjects (41 of 61, 67.2 %) were diagnosed with CVID or hypogammaglobulinemia. Five patients had XLA and one of each had ataxia telangiectasia, severe combined immunodeficiency, IgG subclass deficiency, combined IgG/CD40 deficiency, and hyper IgE syndrome. The diagnosis of 10 subjects was recorded as PID [25].

No acute SBIs were reported. The 95 % confidence interval for the incidence of acute SBIs was 0–0.060. Four patients experienced non-SBIs that commonly occur in PID subjects at a rate of 0.07 infections/patient/year. A total of 224 non-serious infections that were not predefined were also recorded with an incidence of 3.4 infections/subject/year [25].

Fifty-seven subjects were included in the pharmacokinetic study. The median IgG half-life was 35 days (see Table 1). The median trough IgG levels varied from 960 to 1,120 mg/dL. The median IgG C_max_ was 2,050 mg/dL and the median C_min_ was 1,030 mg/dL. A total of 826 infusions of Gammagard Liquid 10 % were administered during the study. The median dose per patient was 455 mg/kg (range 262–710 mg/kg) [25].

Two SAEs (both aseptic meningitis) occurred in one patient and were judged to be related to the study product. Of the 896 non-SAEs, 258 (28.8 %) were possibly or probably related to infusion of the study product. There were 345 temporally associated AEs, i.e., that occurred during or within 72 h of a study infusion. The most frequent temporally associated AEs were headache (22 % of patients), fever (13 % of patients), and fatigue (10 % of patients). Headache was the only AE associated with more than 5 % of infusions (6.9 %) [26].

### Flebogamma^®^ 5 % DIF

Flebogamma^®^ 5 % DIF (dual inactivation and filtration) IVIG was studied in an open-label, historically controlled clinical trial that followed the 2005 FDA Guidance [3].

Flebogamma 5 % DIF was administered to 50 PID patients at doses of 300–600 mg/kg every 21–28 days for 12 months. The patients ranged in age from 15 to 75 years (mean, 38.9 years); 29 (63 %) were male; 35 patients were diagnosed with CVID and 10 with XLA. Thirteen patients were treated every 21 days and 33 were treated every 28 days [27].

Forty-six patients completed the study. All 50 patients received at least one infusion of the study product and were analyzed for safety. One patient experienced one episode of bacterial pneumonia that resulted in an overall SBI rate of 0.021 SBI/patient/year, with an upper 98 % confidence limit of 0.112 SBI/patient/year. Secondary efficacy endpoints are summarized in Table 1. Twenty-three patients (50 %) missed at least 1 day of work/school. The median number of days of work/school missed per subject per year was 0.5 days and the mean was 13 days (range 0–257 days). Twenty-nine patients (63 %) visited a physician or the ER at least once for a visit not scheduled as part of the study [27].

The pharmacokinetics of IgG and specific antibodies were studied in eight patients who were treated every 21 days and 12 who were treated every 28 days. In the 21-day patients, the IgG half-life was 30 ± 9 days, the half-life of five serotypes of *S. pneumoniae* ranged from 14 to 16 days, and the half-life of tetanus antibody was 28 ± 13 days. In the 28-day patients, the IgG half-life was 32 ± 5 days, the half-life of five serotypes of *S. pneumoniae* ranged from 14 to 16 days, and the half-life of tetanus antibody was 24 ± 13 days. IgG trough levels were >900 mg/dL (Table 1) [27].

Forty-three subjects reported at least one AE. There were 144 infusions (20.1 %, 95 % confidence limit 24.4 %) associated with one or more AEs that began within 72 h of infusion. There was one severe AE (hives), but no SAEs. Excluding infections, the most frequent AE was pyrexia (37 % of subjects), headache (35 % of subjects), and wheezing (22 % of subjects) [28].

### Privigen^®^

Utilizing the study design described in the 2005 FDA Guidance, Privigen^®^, a 10 % liquid IVIG, was administered to 80 PID patients. Of these, 59 (73.8 %) were diagnosed with CVID and 21 (26.6 %) with XLA [29]. The study patients, ages 3–69 years, were treated for 12 months with 200–888 mg/kg of Privigen every 21 days (*n* = 16) or 28 days (*n* = 64). Seventy-two patients completed the study (per-protocol population). Of the eight patients who discontinued, four withdrew consent, three discontinued because of AEs, and one died. Six patients experienced an SBI—three cases of pneumonia and one case each of septic arthritis, osteomyelitis, and visceral abscess. The annual SBI rate was 0.08 for the ITT population (upper one-sided 97.5 % confidence limit = 0.182). Sixty-six (82.5 %) patients experienced a total of 255 infections (including SBIs) for an annual rate of 3.55 per patient. The most prevalent infections were sinusitis (31.3 % of patients), nasopharyngitis (22.5 % of patients), and upper respiratory infections (22.5 % of patients) [29, 30].

Fifty-three patients (66.3 %) missed work/school or were unable to perform normal activities because of illness, resulting in an average annual rate of 7.94 days per patient. There were five patients who missed ≥30 days each. Fifteen patients were hospitalized for 166 days (two patients were hospitalized for 116 days), for an annual rate of 2.31 hospitalization days per year [29, 30].

The pharmacokinetics of IgG and IgG subclasses were studied in three patients who were treated every 21 days and 22 patients who were treated every 28 days. The median IgG half-life in all subjects was 36.6 days.

The percentage of infusions with temporally associated AEs related to the study IVIG was 9 %. The percentage of infusions with one or more temporally associated AEs was 20.8 % (upper 97.5 % confidence limit = 23.8 %). Headache (43.8 % of patients), pain (25.0 %), and fatigue (16.3 %) were the AEs reported most often. Sixteen patients (20 %) experienced 38 SAEs. An 11-year-old girl had a hypersensitivity reaction during her second infusion and five SAEs were recorded (hypersensitivity, chills, fatigue, dizziness, and fever). The patient was withdrawn from the study. One patient, diagnosed with lymphoproliferative disorder 6 months into the study, died from associated multisystem organ failure [29, 30].

The direct Coombs test (DAT) changed from negative at baseline to positive after the first or second infusion of the study IVIG in 33 of 77 (42.9 %) patients. No subjects exhibited evidence of hemolytic anemia [22].

### Flebogamma^®^ 10 % DIF

Flebogamma^®^ 10 % DIF was studied in an open-label, historically controlled clinical trial that followed the 2008 FDA Guidance. Subjects with PID were enrolled at six sites in the US and were treated for 12 months with 300–600 mg/kg of the study IVIG every 21 or 28 days. Nineteen patients participated in the pharmacokinetic study of IgG, IgG subclasses, five *S. pneumoniae* serotypes, tetanus antitoxoid, cytomegalovirus (CMV) antibody, and hepatitis B surface antibody (anti-HBV) [31].

One patient suffered one episode of bacterial pneumonia for an annual SBI rate of 0.025/patient/year. The incidences of other efficacy endpoints are listed in Table 1. Non-SBIs documented by positive radiograph or fever occurred in 15 % of patients. Overall, 28 patients reported at least one infectious episode, producing a mean incidence of 2.2 infections per patient per year. Thirty-six patients (78 %) received at least one course of antibiotic therapy and 19 patients (41 %) received at least one course of antibiotic prophylaxis. Twenty subjects (43 %) missed one or more days of work/school/normal activities. Five patients were hospitalized at least once. Twenty-four subjects (52 %) made one or more unscheduled ER or urgent care visits [31, 32].

The half-life of IgG was 34 ± 10 days in the 21-day patients (*n* = 10) and 37 ± 13 days in 28-day patients (*n* = 9). The half-lives of IgG subclasses were similar to that of IgG, but higher variability was observed. For the 21-day subjects, the half-lives of *S. pneumoniae* serotypes 4, 6B, 9V, 14, and 19F ranged from 23 ± 10 to 29 ± 20 days; for the 28-day subjects, the values ranged from 24 ± 6 to 36 ± 33 days. The half-life of antibody to tetanus toxoid was 22 ± 7 days in both groups. The half-lives of antibodies to CMV and HBV were of the same order of magnitude as total IgG [31, 32].

Three patients experienced four SAEs that were unrelated to the study IVIG and one patient experienced four SAEs related to infection. No SAEs were considered to be related to the study IVIG. There were no deaths. Three subjects withdrew from the study because of AEs. Forty-five (98 %) patients experienced one or more AEs irrespective of the relationship to the study product, for a total of 723 AEs. Thirty-eight subjects (83 %) had a product-related AE. Flebogamma 10 % DIF infusions associated with one or more AEs during or within 72 h was 36.7 %, with an upper one-sided 95 % confidence limit of 43.9 %. The most frequent temporal AEs were headache (52 % of subjects), rigors (37 % of subjects), and pyrexia (33 % of subjects) [31, 32].

Forty-three of the 46 subjects enrolled in this study had a negative Coombs test at baseline. Of these, 10 (23.3 %) developed a positive Coombs test during the study. No subjects showed evidence of hemolytic anemia [31, 32].

### Gammaplex^®^

Gammaplex^®^ is a 5 % liquid IVIG that was studied in an open-label clinical trial designed according to the 2005 FDA Guidance. Fifty subjects were enrolled at seven centers; 22 were infused every 21 days and 28 were infused every 28 days. Patients received 279–799 mg/kg of the study IVIG per infusion. Forty-five subjects completed the study. Two patients were discontinued because of an AE, one subject was discontinued because of pregnancy, one was lost to follow up, and another withdrew consent [33].

The patient demographics are shown in Table 1. Six subjects (12 %) had at least one SBI in the 6 months before enrollment. However, there were no SBIs during the study. Thus, the mean SBI rate/year was zero, with an upper one-sided 99 % confidence limit of 0.101. Forty subjects (80 %) had at least one non-serious infection, with a mean rate of 3.28 infections/subject/year. Therapeutic antibiotics were administered to 40 subjects (annual rate of 47.2 days/subject/year). Thirty-three courses of prophylactic antibiotics were given to 16 patients, seven of whom were on prophylactic antibiotics throughout the entire study. Twenty-three patients (46 %) missed 394 days of work or school because of infection or other illness, for an annual rate of 8.73 days/patient/year [34]. Four patients were hospitalized for 29 days, producing an annual rate of 0.75 hospitalization days/subject/year [33].

All 50 subjects had at least one AE. Twenty-four subjects (48 %) had a product-related AE. The 21-day subjects had more product-related AEs (14/22, 64 %) than the 28-day subjects (10/28, 36 %). The total number of temporally associated AEs (0–72 h) was 237 (0.34 AEs per infusion). Of the 703 infusions administered, 21 % were associated with one or more temporally associated AEs. The upper one-sided 97.5 % confidence limit was 24 %. Of the 237 temporally associated AEs, 115 (49 %) were judged to be product-related. Headache was the most common product-related AE (7.5 % of 703 infusions) [33].

Five subjects (10 %) experienced seven SAEs. Two of the SAEs were considered to be related to the infusion of Gammaplex (thrombosis and chest pain) [33].

Forty-seven of the 50 subjects enrolled in this study had a negative DAT at baseline. Four subjects (8.5 %) developed a positive DAT during the study. No subjects showed evidence of hemolytic anemia [33].

Pharmacokinetics were assessed in nine subjects on the 21-day infusion schedule and 15 subjects on the 28-day infusion schedule. The IgG half-life was 42 ± 26 days in the 21-day patients and 41 ± 14 days in the 28-day patients [34].

### Biotest-IVIG

Biotest-IVIG, a 10 % liquid IVIG, was studied in an open-label, phase III trial that followed the 2005 FDA Guidance. The primary efficacy endpoint was to demonstrate that the rate of acute SBIs was <1.0 per person/year. Secondary efficacy parameters included infections of any kind or severity, time to the first infection of any kind, time to first SBI, days missed from school or work due to infection, days on antibiotics, hospitalizations, and days of hospitalization due to infection [35].

Sixty-three patients were enrolled, treated with 746 infusions of Biotest-IVIG (300–800 mg/kg), and included in the population evaluated for safety. Seventeen patients were infused every 3 weeks and 46 were infused every 4 weeks. Of the 63 enrollees, 51 patients completed the study. The most common immunodeficiency diagnosis was CVID (81 % of patients). The average age was 41.2 years (range 6–75 years). Eight patients had a history of acute SBIs prior to study entry [35].

Two SBIs occurred during the 53.54 person-years of the study, resulting in an incidence of 0.037 SBI/patient/year, with an upper 99 % confidence limit of ≤0.101 SBI/patient/year. Twenty-one patients (33 %) missed 122 days of work or school because of infection, resulting in a rate of 2.28 days/patient/year. Thirty-eight patients (60 %) were treated with therapeutic antibiotics for 1,812 days, for a rate of 39.1 days of therapeutic antibiotics/patient/year [35].

A total of 937 AEs were recorded in the 63 patients in the safety population. 339 (36 %) of the AEs occurred in six patients. Fifty-nine patients (94 %) reported at least one AE. Of these, 40 patients (64 %) experienced an AE that was related to study product. The most frequent severe AEs considered to be related to the study drug were headache (3 patients, 4.8 %) and migraine and fatigue (2 patients, 3.2 % each). Eleven SAEs were reported in seven (11 %) patients. Two of the SAEs (vomiting and dehydration) that lead to the hospitalization of one patient were considered to be related to the study product. There were no deaths [35].

There were 431 temporally associated AEs in 47 (75 %) patients. The proportion of infusions with one or more temporally associated AEs, regardless of the relationship to the study product, was 27.7 %, with an upper one-sided 95 % confidence limit of ≤30.6 %. The most frequent temporally associated AE was headache (115 infusions in 27 patients), followed by fatigue (59 infusions in 15 patients) [35].

The pharmacokinetics population was composed of 5 patients in the 3-week infusion group and 16 patients in the 4-week group. The IgG half-life for the total population was 30 ± 11 days, with a range of 16.2–51.6 days. The half-lives of specific antibodies ranged from 25 to 84 days (anti-*H. influenzae* b and anti-tetanus, respectively.) The half-lives of three antibodies to *S. pneumoniae* serotypes were 41, 60, and 30 days [35].

The mean trough IgG levels were 1,076 ± 254 mg/dL (range 606–1,780 mg/dL) in the 21-day patients and 943 ± 215 (range 487–2,250 mg/dL) in the 28-day patients. The 487 mg/dL trough value was a single test result reported at the sixth infusion in one patient [35].

## Discussion

The 1999 BPAC meeting concluded that each IVIG is unique and should not be treated as a single generic product [1]. Consequently, the FDA proposed that phase III clinical trials of new IVIGs (including licensed products with modified manufacturing procedures) should be more rigorous than previous trials of small numbers of patients [18–20]. It soon became apparent that the number of patients with primary immunodeficiency that could be recruited for IVIG clinical trials was too limited to support the number of new IVIGs that needed to be tested. This prompted the analysis of possible IVIG trials that would reduce the sample size.

A new proposal was to conduct a single-arm, 12-month, open-label study with comparison to historical controls for safety, pharmacokinetics, and efficacy endpoints. The FDA defined infusional AEs as those that are temporally associated with an IVIG infusion, i.e., occurred during or within 72 h of an infusion. The safety endpoint was defined as the proportion of infusions with one or more temporally associated AEs (including AEs that are determined not to be product-related). The target for this endpoint is an upper one-sided 95 % confidence limit of <0.40 [4].

The efficacy endpoint is the incidence of SBIs. Based on historical data, patients with primary humoral immunodeficiency experienced approximately 4 or more SBIs per year prior to routine immunoglobulin therapy [4]. Also, the SBI rate was <0.5 per year during periods of regular immunoglobulin therapy (200–600 mg/kg/infusion, every 3 or 4 weeks) [4]. Accordingly, the FDA set an SBI target of an upper one-sided 99 % confidence limit <1.0 SBI per person per year. The criteria for diagnosing SBIs were defined in the Guidance [4].

The safety and efficacy endpoints in the FDA IVIG Guidances [3, 4] have essentially been incorporated into each of the IVIG clinical trials published since 2004. The trial that compared Gamunex 10 % to Gamimune N 10 % used serious infection endpoints that were different from the other studies.

Although many of the same study sites were involved in studies of multiple products, the patient population in each clinical trial is assumed to be different. However, the majority (52 to 92 %) of patients in each study were diagnosed with CVID. The proportion of XLA patients varied from 0 to 28 % (Table 1).

The rate of SBIs (the primary efficacy endpoint) in each study was well below the 0.5 per year reported by the FDA as the historical frequency during periods of regular immunoglobulin therapy [4]. SBI rates varied from 0 to 0.08 SBI/patient/year (Table 1). The rate of non-serious infections also varied from study to study, with values of 1.96–3.55 infections/patient/year (Table 1).

Days of work and school missed because of PID-related illness were reported in six studies and varied from an average of 2.1 days/year (Biotest-IVIG) to 13 days/year (Flebogamma 5 %). The values of 13 and 8.7 days of missed work/school resulted from relatively few patients. The average number of hospitalization days per year caused by PID-related infections was low in each study, ranging from 0.21 (Biotest-IVIG) to 2.31 (Privigen). The studies that recorded unscheduled physician or ER visits as an efficacy parameter reported mean values of 2 visits/patient/year (Octagam) to 5.6 visits/patient/year (Gammaplex).

Antibiotic administration was reported in four studies (Table 1). Therapeutic antibiotic days/patient/year were very similar for the three studies that reported it. Prophylactic antibiotics were reported as 81 days/patient/year in the Flebogamma 5 % study and 46 days/patient/year in the Flebogamma 10 % study. Two studies reported only the total number of antibiotic days/patient/year—Privigen (87.4 days) and Biotest-IVIG (82 days). Total antibiotic days/patient/year in the Flebogamma 5 % and Flebogamma 10 % studies were 136.7 and 103.8, respectively.

IgG half-life values ranged from 20 ± 4 days in the Biotest-IVIG 21-day infusion group to 37 ± 13 days in the Flebogamma 10 % 28-day infusion group. IgG half-lives reported without segregation into infusion groups ranged from 34 to 41 ± 17 days. When recorded, the half-lives of IgG subclasses and specific antibodies were the essentially the same as the total IgG. The lack of standardized test procedures for determining specific antibody concentrations renders the comparison of antibody trough and other pharmacokinetic parameters difficult.

The reporting of AEs varied from study to study, with temporally associated AEs varying from 30 min to 72 h post-infusion. Using the 72-h time frame, the incidence of infusions with one or more temporal AEs (regardless of causality) produced AE rates of 20.1–47 % (Table 2). AEs considered to be related to the study IVIG ranged from 5 % of infusions for Octagam to 27.6 % of Flebogamma 10 % infusions (Table 2). Headache was the most common AE in every study.

The appearance of Coombs positivity after infusion of the study IVIGs occurred in four clinical trials. The proportion of patients that became Coombs-positive after infusion varied from 8.5 to 47 %. However, none of the patients in any of the trials developed evidence of hemolysis or anemia.

Clinical results with the newest IVIG from Biotest compared favorably to the results obtained with other IVIGs. Investigators observed a relatively low incidence of non-serious infections (2.6 per patient per year), few missed days of work/school (2.28 per patient per year), and a low rate of hospitalizations (0.21 per patient per year). The number of days of antibiotic treatment during the Biotest-IVIG study (82 days) were also lower than reports from other studies (range 87.4–136.7 days). AE rates were comparable to the rates reported for other products.

In summary, a review of IVIG clinical trial results reported in the new millennium show that clinical trial protocols are becoming more and more standardized, thanks to efforts of the FDA. Although comparisons should be conducted cautiously, the standardization of clinical trial data offers the opportunity to compare results from study to study. If standardized tests for quantifying specific antibodies are developed, pharmacokinetic studies may provide more meaningful information with respect to appropriate IVIG doses to prevent infection.
